# Weighing the Risk of Seizure Control: A Case of Levetiracetam-Induced Rhabdomyolysis

**DOI:** 10.7759/cureus.74111

**Published:** 2024-11-20

**Authors:** Sierra Lyles, Rediet Tefera Atalay, Shay Taylor, Girma M Ayele, Samrawit W Zinabu, Ahmad Mohammed, Miriam B Michael, Vishal A Poddar

**Affiliations:** 1 Neurology, Howard University Hospital, Washington, DC, USA; 2 Internal Medicine, Howard University Hospital, Washington, DC, USA; 3 Internal Medicine, University of Maryland, Baltimore, USA; 4 Pulmonology and Critical Care, Howard University Hospital, Washington, DC, USA

**Keywords:** creatine kinase, levetiracetam, rhabdomyolysis, seizure, seizure medications

## Abstract

Rhabdomyolysis, a severe condition marked by the breakdown of muscle tissue, leads to the release of intracellular contents into the bloodstream. This condition can be triggered by a range of factors, including intense physical activity, traumatic injuries, certain medications, and infections. Diagnosis typically involves detecting elevated creatine phosphokinase (CPK) levels alongside characteristic clinical symptoms. Levetiracetam-induced rhabdomyolysis is an exceptionally rare phenomenon, with only a few cases documented in the literature. In this report, we present a 47-year-old male patient in our intensive care unit who developed rhabdomyolysis after continuing his home dose of levetiracetam following a witnessed seizure. Despite four days of aggressive hydration, his CPK levels continued to rise, ultimately peaking at 46,671 U/L. With no other apparent causes, levetiracetam was suspected as the culprit and subsequently discontinued. Remarkably, the patient's condition improved quickly after stopping the medication, with CPK levels dropping within two days, allowing for a successful transition to lacosamide. Although rare, this case highlights the critical need to monitor CPK levels in patients who develop rhabdomyolysis symptoms after restarting levetiracetam therapy. We recommend considering discontinuation of levetiracetam if no other identifiable causes are found.

## Introduction

Initially, levetiracetam was comparable to other antiepileptic drugs (AEDs) in terms of seizure control and even more so for its low risk of side effects. Among the minimal side effects were headaches, nausea, dizziness, and drowsiness. Rare instances of levetiracetam-induced rhabdomyolysis have been documented in a limited number of case reports, typically in patients who had previously tolerated the medication well [1]. Compared to other AEDs, levetiracetam had the lowest risk of treatment-emergent adverse effects at all doses, including the highest effective recommended daily dose. Adverse events being significantly lower in levetiracetam use also contributed to its lowest withdrawal rate among other treatment options [2]. Recent studies have shown a shift in its ability to dominate all adverse effects, mainly rhabdomyolysis. While rhabdomyolysis is still a rare occurrence over all AEDs with only 1,142 cases in the overall 261,586 reported adverse effects, 36.9% of them were from levetiracetam [3]. With new data showing the heightened occurrence with levetiracetam use, watching out for classic signs may help diagnose effectively in those rare cases.

Rhabdomyolysis is characterized by the breakdown of skeletal muscle fibers, causing the release of cellular contents such as creatine kinase (CK) and myoglobin into the bloodstream. Therefore, the most sensitive serological indicator of rhabdomyolysis is an elevated serum CK level [4]. In most cases, an elevated CK can be sufficient to diagnose rhabdomyolysis due to the absence of expected symptoms such as weakness or muscle pain [5].

This case report highlights a 47-year-old male patient admitted to the medical intensive care unit (MICU) due to a seizure disorder following drug discontinuation. Reintroduction of levetiracetam resulted in rhabdomyolysis leading to consistently rising creatine phosphokinase (CPK) in the absence of any other identifiable cause.

## Case presentation

This case involves a 47-year-old man with a medical history of epilepsy secondary to a traumatic brain injury resulting in bifrontal encephalomalacia. He was presented to the emergency department (ED) by emergency medical service after experiencing a witnessed seizure. At the scene, he received Versed before being transported to the ED, where he subsequently required intubation for airway protection due to altered mental status and concerns of aspiration pneumonia. With his current presentation, he was admitted to the hospital as a MICU patient but remained in the ED because of bed availability. On the second day in the ED, hospital admission day 1, the patient was successfully extubated. Comparably, he had improved clinically and had no complaints. He was then restarted on levetiracetam, one of his prescribed home medications, with an initial loading dose before titrating up to a dose of 1 g every 12 hours per neurology recommendation. While available prior to the hospitalization, he was non-compliant with his home levetiracetam and was not on any other medications. It was unclear how long he was prescribed levetiracetam prior to stopping, but the patient and family denied any previous adverse effects.

**Figure 1:**
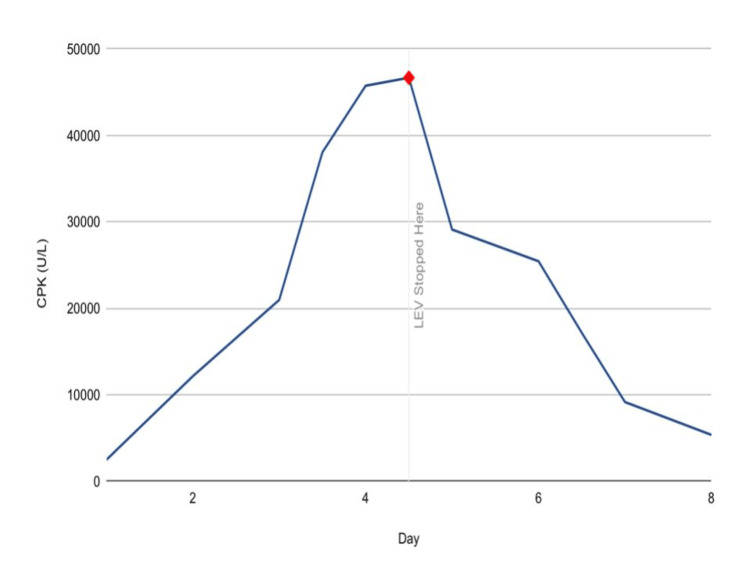
CPK trend CPK: creatine phosphokinase; LEV: levetiracetam

On hospital admission day two, three days after the initial presentation, it was noted that his renal function was falling, leading to further investigation, which revealed increasing CPK levels (Figure 1) alongside elevated myoglobin, aspartate transaminase (AST), and alanine transaminase (ALT) levels.

Despite extensive testing, including urine toxicology screening and negative results for ethanol, antinuclear antibody (ANA), thyroid-stimulating hormone (TSH), blood cultures, *Mycoplasma pneumoniae* antibodies, and respiratory panel, the cause of elevated CPK levels remained unclear. Aggressive intravenous fluid therapy was initiated following the elevated CPK. Initially, the patient received 500 mL/hr of normal saline for the first six hours, followed by a reduced rate of 300 mL/hr for the subsequent two days. Despite adequate urine output, the CPK levels continued to rise and peaked at a level of 46,671 U/L on day four of administration. The MICU team consulted neurology for a concern of a potential association of rhabdomyolysis with levetiracetam. While the dose was adjusted based on renal functions, we were unable to obtain labs on the levetiracetam levels. The clinical association was made between restarting levetiracetam, when the patient was clinically stable, and the progression to renal failure. This led to the decision to discontinue levetiracetam and initiate lacosamide, which was quickly validated with the improvement of his renal function. After discontinuing levetiracetam, the patient's CPK levels decreased dramatically, returning to baseline nearly as soon as it rose. His latest CPK level was recorded at 5,340 U/L, with liver function tests showing improvement without the need for dialysis.

## Discussion

Levetiracetam, approved in November 1999, is commonly used as an adjunctive therapy for partial-onset seizures in adults aged 16 years and older. When combined with other AEDs, the most frequently reported adverse events are central nervous system-related, typically mild to moderate in severity. These side effects predominantly occur within the first four weeks of treatment. Notably, well-controlled clinical trials within the recommended dose range of 1,000-3,000 mg/day did not establish a clear dose-response relationship for these adverse events [6].

Structurally and mechanistically, levetiracetam distinguishes itself from other AEDs. It binds to synaptic vesicle protein 2A, inhibits calcium release, counteracts negative modulators of gamma-aminobutyric acid (GABA) and glycine currents, and inhibits N-type calcium channels [7]. Levetiracetam is rapidly absorbed, highly bioavailable, minimally metabolized, and primarily eliminated through the kidneys. Significantly, it does not induce cytochrome P450 enzymes, minimizing the risk of significant drug interactions, even with other AEDs [8].

Despite its widespread use, rhabdomyolysis is rarely associated with AEDs, but levetiracetam is one to be mindful of. Interestingly, while levetiracetam’s primary binding site is synaptic vesicle protein 2A in the brain, it also binds to SV2A proteins found in muscle tissue, the parathyroid, and fibroblasts [9]. The precise mechanism behind levetiracetam-induced rhabdomyolysis remains unclear. One theory suggests that levetiracetam may interact with SV2A in the motor nerve terminals of slow muscle fibers, leading to increased cholinergic neurotransmission and subsequent muscle stress, ultimately contributing to rhabdomyolysis [5].

A 2020 study reviewed cases of levetiracetam-induced rhabdomyolysis from 2014 to 2019. The majority of cases occurred in individuals aged 19-30, with only one case reported in the over-60 age group. CPK levels ranged from 1,368 IU/L to 49,539 U/L, with elevations occurring within 12-36 hours of treatment initiation and peaking within three to five days. The study suggested that variations in CPK levels might be influenced by differences in muscle mass among patients [4]. However, a review of the literature has not identified any specific predisposing factors for levetiracetam-induced rhabdomyolysis.

Another case series evaluated in 2023 examining four patients saw that rhabdomyolysis developed 1-15 days after levetiracetam was initiated, with pathognomonic serum CPK levels rising despite appropriate hydration. In each case, CPK would continue to rise and peak until switched to another AED. While these patients also experienced post-octal periods, due to the duration of CPK elevation, timing of rhabdomyolysis after initiating levetiracetam, and prompt improvement after discontinuation, levetiracetam was considered the cause of their symptoms [10]. This case series nearly mirrors our case, showing that rhabdomyolysis with levetiracetam has a common progression that may be helpful in diagnosis.

Limitations

Common to other case reports, this study was done in a retrospective manner, looking at the patient's chart as our method of gathering information. Due to this, certain facts that may have been elicited through the interview could not be obtained. While the information presented from the chart review is accurate to the sequence of events, being unable to quote the patient takes out additional personal information the patient may want to be shared. Creating a case series or a systematic review can lead to further advances in research by assessing common factors between individuals, building a greater sense of the pathogenesis.

## Conclusions

Levetiracetam has established itself as a widely utilized AED, owing to its distinctive structure, mechanism of action, and limited drug interactions. Although rhabdomyolysis remains an uncommon side effect, the exact mechanism behind it is still unclear. Evidence points to a higher prevalence of levetiracetam-induced rhabdomyolysis in younger adults, with fluctuations in CPK levels possibly reflecting variations in muscle mass. Importantly, rhabdomyolysis can manifest in both long-term users of levetiracetam and those newly introduced to the medication. Further studies should focus on causative relationships between levetiracetam use and rhabdomyolysis, assessing factors such as dose-dependent toxicities, of which there is no substantial data. This report underscores the importance of monitoring CPK levels during the initiation or reintroduction of levetiracetam, as rhabdomyolysis remains a possible complication. Clinicians should remain vigilant, recognizing that even patients with prior tolerance to levetiracetam are not exempt from this risk, and rhabdomyolysis should be included in differential diagnoses during clinical evaluations.
